# P44 - Is travelling with commercial airplanes dangerous for peanut allergic children? Results of an air travel simulating provocation model

**DOI:** 10.1186/2045-7022-4-S1-P99

**Published:** 2014-02-28

**Authors:** Søren Wille

**Affiliations:** 1 Helsingborg Hospital, Helsingborg, Sweden

## 

Peanut allergy is increasing Food allergy has a huge impact on Quality of Life for the patients and their families. Allergy is seldom reported as emergencies from Commercial Airlines but many peanut allergic patients’ reports symptoms during air flight by ingestion of peanuts but mostly by inhalation.

Many peanut allergic patients avoid travelling by airplane. The risk for reaction is controversial.

From opening peanut bags and stirring in peanut bowl. No severe reaction was observed. 23 children were tested. Nine patients had a mild reaction like itching of skin, eyes or mouth or urticaria. Three patients received treatment with antihistamine and one with oral steroid. Only one had objectively observed symptoms.

Air travel seems to be safe with only risk of mild symptoms in peanut allergic patient even when peanuts are served during flight.

